# Dichlorido{2-[(thio­phen-2-ylmeth­yl)imino­meth­yl]pyridine-κ^2^ *N*,*N*′}palladium(II)

**DOI:** 10.1107/S1600536811037214

**Published:** 2011-09-17

**Authors:** Martin O. Onani, William M. Motswainyana

**Affiliations:** a University of the Western Cape, Cape Town, Bellville 7535, South Africa

## Abstract

In the title compound, [PdCl_2_(C_11_H_10_N_2_S)], the Pd^II^ ion is four-coordinated in a distorted square-planar environment by two N atoms of the chelating 2-[(thio­phen-2-ylmeth­yl)imino­meth­yl]pyridine ligand and two chloride anions. The thio­phene ring is rotationally disordered over two orientations in a 1:1 ratio. The crystal packing exhibits weak inter­molecular C—H⋯Cl and C—H⋯S hydrogen bonds.

## Related literature

For the synthesis of imino­pyridyl ligands and their transition metal-based complexes, see: Zhang *et al.* (2006 ▶); Bianchini *et al.* (2010 ▶). For related structures, see: Doherty *et al.* (2002 ▶); Ojwach *et al.* (2009 ▶); Motswainyana *et al.* (2011 ▶). For similar structures with nickel, see: Britovsek *et al.* (2003 ▶).
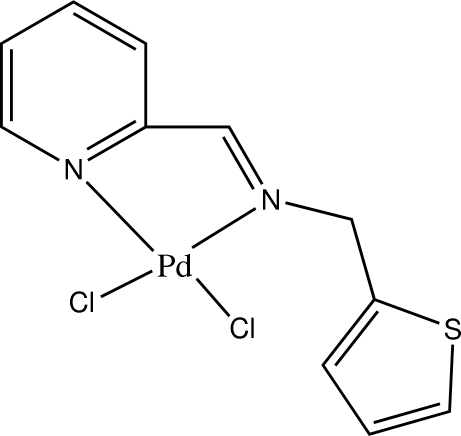


## Experimental

### 

#### Crystal data

[PdCl_2_(C_11_H_10_N_2_S)]
*M* *_r_* = 379.57
Monoclinic, 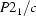

*a* = 8.0061 (19) Å
*b* = 17.768 (4) Å
*c* = 8.864 (2) Å
β = 98.353 (3)°
*V* = 1247.6 (5) Å^3^
*Z* = 4
Mo *K*α radiation
μ = 2.06 mm^−1^
*T* = 100 K
0.24 × 0.19 × 0.06 mm

#### Data collection

Bruker SMART APEX CCD area-detector diffractometer
Absorption correction: multi-scan (*SADABS*; Bruker, 2009 ▶) *T*_min_ = 0.638, *T*_max_ = 0.887
6411 measured reflections
2656 independent reflections
2238 reflections with *I* > 2σ(*I*)
*R*_int_ = 0.036

#### Refinement

*R*[*F* ^2^ > 2σ(*F* ^2^)] = 0.042
*wR*(*F* ^2^) = 0.144
*S* = 1.07
2656 reflections
149 parameters
26 restraints
H-atom parameters constrained
Δρ_max_ = 0.96 e Å^−3^
Δρ_min_ = −0.98 e Å^−3^

### 

Data collection: *APEX2* (Bruker, 2009 ▶); cell refinement: *SAINT* (Bruker, 2009 ▶); data reduction: *SAINT*; program(s) used to solve structure: *SHELXS97* (Sheldrick, 2008 ▶); program(s) used to refine structure: *SHELXL97* (Sheldrick, 2008 ▶); molecular graphics: *X-SEED* (Barbour, 2001 ▶; Atwood & Barbour, 2003 ▶); software used to prepare material for publication: *SHELXL97*.

## Supplementary Material

Crystal structure: contains datablock(s) I, global. DOI: 10.1107/S1600536811037214/cv5147sup1.cif

Structure factors: contains datablock(s) I. DOI: 10.1107/S1600536811037214/cv5147Isup2.hkl

Additional supplementary materials:  crystallographic information; 3D view; checkCIF report

## Figures and Tables

**Table 1:** Hydrogen-bond geometry (Å, °)

*D*—H⋯*A*	*D*—H	H⋯*A*	*D*⋯*A*	*D*—H⋯*A*
C12—H12⋯Cl15^i^	0.95	2.70	3.508 (6)	143
C6*B*—H6*B*⋯Cl16^ii^	0.95	2.74	3.622 (14)	155
C7*A*—H7*A*⋯S8*A*^iii^	0.95	2.69	3.468 (12)	139

Symmetry codes: (i) 
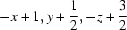
; (ii) 
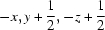
; (iii) 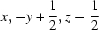.

**Fig. 1.**
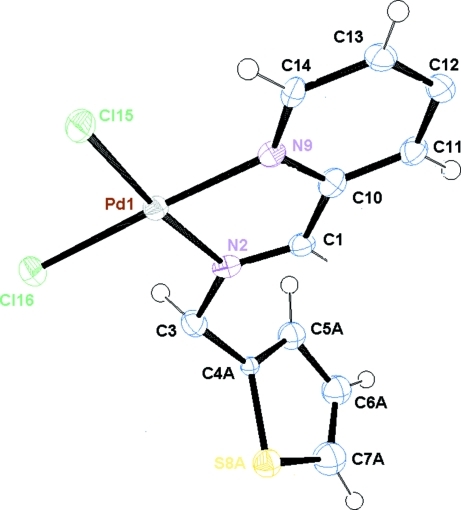
The molecular structure of (I) showing atomic numbering and 50% probability displacement ellipsoids. For the rotationally disordered thiophene ring only one orientation is shown.

## supplementary crystallographic information

### Comment

Nitrogen based ligands have attracted considerable interest due to their stability and various activities (Bianchini *et al*., 2010; Zhang *et al.*, 2006). These ligands can be complexed to various transition metals to form stable metal complexes which exhibit different colours and geometries (Bianchini *et al.*, 2010; Britovsek *et al.*, 2003; Motswainyana *et al.*, 2011). Herewith we present the crystal stucture of the title compound (I).

The asymmetric unit of (I) contains one molecule of the Pd^II^ complex (Fig 1). All bond lengths and angles are normal and comparable with those observed in the related complexes (Zhang *et al*., 2006; Motswainyana *et al.*, 2011; Doherty *et al.*, 2002). Pd^II^ ion has a distorted square planar environment being coordinated by the *N,N'*-bidentate ligand and two Cl anios. The Pd – Cl bond length *trans* to the pyridyl N atom is slightly longer than the Pd – Cl bond length *trans* to the amine N atom showing the stronger *trans* influence of the pyridyl group compared to the secondary amine.

The crystal packing exhibits weak intermolecular C—H···Cl and C—H···S hydrogen bonds (Table 1).

### Experimental

To a solution of [PdCl_2_(cod)] (0.10 g, 0.35 mmol) in CH_2_Cl_2_ (15 ml) was added a solution of 1-phenyl-*N*-(2-thienylmethyl)methanimine (0.07 g, 0.35 mmol) in CH_2_Cl_2_ (5 ml). The solution was stirred for 6 h to give a light yellow precipitate. The precipitate was filtered to obtain a light yellow solid. Recrystallization from a mixture of CH_2_Cl_2_: hexane solution afforded single crystals suitable for X-ray analysis. Yield = 0.110 g (85%).

### Refinement

All hydrogen atoms were placed at idealized positions with d(C—H) = 0.95-0.99 Å and refined as riding on their parent atoms, with *U*_iso_ (H) = 1.2 – 1.5 *U*_eq_ (C). The thiophene ring was treated as rotationally disordered over two orientations in a ratio 1:1.

### Crystal data

**Table:**

[PdCl_2_(C_11_H_10_N_2_S)]	*F*(000) = 744
*M**_r_* = 379.57	*D*_x_ = 2.021 Mg m^−^^3^
Monoclinic, *P*2_1_/*c*	Mo *K*α radiation, λ = 0.71073 Å
Hall symbol: -P 2ybc	Cell parameters from 3002 reflections
*a* = 8.0061 (19) Å	θ = 2.3–27.6°
*b* = 17.768 (4) Å	µ = 2.06 mm^−^^1^
*c* = 8.864 (2) Å	*T* = 100 K
β = 98.353 (3)°	Plate, yellow
*V* = 1247.6 (5) Å^3^	0.24 × 0.19 × 0.06 mm
*Z* = 4	

### Data collection

**Table:**

Bruker SMART APEX CCD area-detector diffractometer	2656 independent reflections
Radiation source: fine-focus sealed tube	2238 reflections with *I* > 2σ(*I*)
graphite	*R*_int_ = 0.036
ω scans	θ_max_ = 27.8°, θ_min_ = 2.3°
Absorption correction: multi-scan (*SADABS*; Bruker, 2009)	*h* = −9→9
*T*_min_ = 0.638, *T*_max_ = 0.887	*k* = −22→22
6411 measured reflections	*l* = −11→8

### Refinement

**Table:**

Refinement on *F*^2^	Primary atom site location: structure-invariant direct methods
Least-squares matrix: full	Secondary atom site location: difference Fourier map
*R*[*F*^2^ > 2σ(*F*^2^)] = 0.042	Hydrogen site location: inferred from neighbouring sites
*wR*(*F*^2^) = 0.144	H-atom parameters constrained
*S* = 1.07	*w* = 1/[σ^2^(*F*_o_^2^) + (0.096*P*)^2^] where *P* = (*F*_o_^2^ + 2*F*_c_^2^)/3
2656 reflections	(Δ/σ)_max_ = 0.017
149 parameters	Δρ_max_ = 0.96 e Å^−^^3^
26 restraints	Δρ_min_ = −0.98 e Å^−^^3^

### Special details

**Table:**

Geometry. All e.s.d.'s (except the e.s.d. in the dihedral angle between two l.s. planes) are estimated using the full covariance matrix. The cell e.s.d.'s are taken into account individually in the estimation of e.s.d.'s in distances, angles and torsion angles; correlations between e.s.d.'s in cell parameters are only used when they are defined by crystal symmetry. An approximate (isotropic) treatment of cell e.s.d.'s is used for estimating e.s.d.'s involving l.s. planes.
Refinement. Refinement of *F*^2^ against ALL reflections. The weighted *R*-factor *wR* and goodness of fit *S* are based on *F*^2^, conventional *R*-factors *R* are based on *F*, with *F* set to zero for negative *F*^2^. The threshold expression of *F*^2^ > σ(*F*^2^) is used only for calculating *R*-factors(gt) *etc*. and is not relevant to the choice of reflections for refinement. *R*-factors based on *F*^2^ are statistically about twice as large as those based on *F*, and *R*- factors based on ALL data will be even larger.

### Fractional atomic coordinates and isotropic or equivalent isotropic displacement parameters (Å^2^)

**Table:**

	*x*	*y*	*z*	*U*_iso_*/*U*_eq_	Occ. (<1)
Pd1	0.30571 (5)	−0.01089 (2)	0.58087 (4)	0.01686 (18)	
Cl15	0.40550 (17)	−0.08117 (7)	0.79082 (15)	0.0254 (3)	
Cl16	0.16329 (16)	−0.11300 (7)	0.46964 (15)	0.0238 (3)	
N2	0.2291 (5)	0.0612 (2)	0.4078 (5)	0.0179 (9)	
N9	0.4236 (5)	0.0838 (2)	0.6682 (5)	0.0167 (8)	
C1	0.2820 (6)	0.1286 (3)	0.4327 (6)	0.0186 (10)	
H1	0.2521	0.1669	0.3590	0.022*	
C3	0.1145 (6)	0.0400 (3)	0.2666 (6)	0.0211 (10)	
H3A	0.1563	−0.0078	0.2285	0.025*	
H3B	0.0016	0.0298	0.2950	0.025*	
C10	0.3879 (6)	0.1454 (3)	0.5747 (6)	0.0208 (10)	
C11	0.4519 (6)	0.2153 (3)	0.6190 (6)	0.0218 (11)	
H11	0.4243	0.2577	0.5548	0.026*	
C12	0.5564 (7)	0.2239 (3)	0.7569 (6)	0.0225 (11)	
H12	0.6009	0.2720	0.7881	0.027*	
C13	0.5949 (7)	0.1613 (3)	0.8484 (6)	0.0219 (10)	
H13	0.6690	0.1653	0.9420	0.026*	
C14	0.5223 (6)	0.0922 (3)	0.8001 (6)	0.0203 (10)	
H14	0.5449	0.0496	0.8647	0.024*	
S8A	−0.0145 (4)	0.1806 (2)	0.1442 (4)	0.0239 (7)*	0.50
C4A	0.095 (2)	0.0978 (8)	0.1389 (14)	0.024 (9)*	0.50
C5A	0.1525 (17)	0.0901 (7)	−0.0021 (15)	0.029 (4)*	0.50
H5A	0.2128	0.0473	−0.0287	0.035*	0.50
C6A	0.1126 (18)	0.1522 (8)	−0.1017 (14)	0.018 (4)*	0.50
H6A	0.1439	0.1564	−0.2007	0.021*	0.50
C7A	0.0239 (15)	0.2047 (6)	−0.0361 (13)	0.016 (3)*	0.50
H7A	−0.0137	0.2506	−0.0848	0.019*	0.50
S8B	0.1827 (5)	0.07989 (19)	−0.0198 (4)	0.0248 (8)*	0.50
C4B	0.093 (3)	0.0937 (10)	0.1403 (18)	0.011 (12)*	0.50
C5B	0.0011 (15)	0.1601 (7)	0.1324 (13)	0.025 (3)*	0.50
H5B	−0.0583	0.1773	0.2111	0.030*	0.50
C6B	0.0055 (17)	0.1999 (8)	−0.0075 (15)	0.030 (4)*	0.50
H6B	−0.0496	0.2466	−0.0327	0.036*	0.50
C7B	0.098 (2)	0.1628 (9)	−0.0980 (19)	0.037 (6)*	0.50
H7B	0.1144	0.1804	−0.1959	0.045*	0.50

### Atomic displacement parameters (Å^2^)

**Table:**

	*U*^11^	*U*^22^	*U*^33^	*U*^12^	*U*^13^	*U*^23^
Pd1	0.0192 (3)	0.0166 (3)	0.0149 (3)	0.00044 (13)	0.00286 (17)	0.00049 (13)
Cl15	0.0346 (7)	0.0195 (6)	0.0212 (7)	0.0002 (5)	0.0014 (5)	0.0049 (5)
Cl16	0.0279 (7)	0.0199 (6)	0.0233 (7)	−0.0034 (5)	0.0028 (5)	−0.0032 (5)
N2	0.016 (2)	0.024 (2)	0.014 (2)	0.0025 (17)	0.0029 (16)	0.0014 (16)
N9	0.016 (2)	0.019 (2)	0.015 (2)	0.0022 (15)	0.0009 (16)	0.0031 (15)
C1	0.022 (3)	0.020 (2)	0.014 (2)	0.0006 (19)	0.0036 (19)	0.0013 (18)
C3	0.021 (3)	0.021 (2)	0.022 (3)	−0.002 (2)	0.002 (2)	−0.003 (2)
C10	0.017 (2)	0.023 (2)	0.023 (3)	0.0010 (19)	0.004 (2)	0.003 (2)
C11	0.024 (3)	0.022 (2)	0.019 (3)	−0.001 (2)	0.003 (2)	0.004 (2)
C12	0.028 (3)	0.018 (2)	0.021 (3)	0.000 (2)	0.005 (2)	−0.0017 (19)
C13	0.023 (3)	0.027 (3)	0.015 (2)	−0.002 (2)	−0.0009 (19)	−0.001 (2)
C14	0.021 (3)	0.019 (2)	0.022 (3)	−0.0049 (19)	0.006 (2)	0.003 (2)

### Geometric parameters (Å, °)

**Table:**

Pd1—N2	2.024 (4)	C13—C14	1.400 (7)
Pd1—N9	2.027 (4)	C13—H13	0.9500
Pd1—Cl15	2.2866 (13)	C14—H14	0.9500
Pd1—Cl16	2.2885 (13)	S8A—C4A	1.715 (13)
N2—C1	1.279 (6)	S8A—C7A	1.725 (11)
N2—C3	1.489 (6)	C4A—C5A	1.402 (14)
N9—C14	1.321 (7)	C5A—C6A	1.420 (14)
N9—C10	1.378 (6)	C5A—H5A	0.9500
C1—C10	1.443 (7)	C6A—C7A	1.354 (14)
C1—H1	0.9500	C6A—H6A	0.9500
C3—C4B	1.462 (13)	C7A—H7A	0.9500
C3—C4A	1.519 (12)	S8B—C4B	1.698 (14)
C3—H3A	0.9900	S8B—C7B	1.726 (14)
C3—H3B	0.9900	C4B—C5B	1.388 (15)
C10—C11	1.379 (7)	C5B—C6B	1.432 (14)
C11—C12	1.386 (7)	C5B—H5B	0.9500
C11—H11	0.9500	C6B—C7B	1.340 (15)
C12—C13	1.385 (7)	C6B—H6B	0.9500
C12—H12	0.9500	C7B—H7B	0.9500
			
N2—Pd1—N9	80.64 (16)	C12—C13—C14	118.6 (5)
N2—Pd1—Cl15	173.71 (12)	C12—C13—H13	120.7
N9—Pd1—Cl15	93.10 (12)	C14—C13—H13	120.7
N2—Pd1—Cl16	95.62 (13)	N9—C14—C13	122.6 (5)
N9—Pd1—Cl16	176.22 (11)	N9—C14—H14	118.7
Cl15—Pd1—Cl16	90.63 (5)	C13—C14—H14	118.7
C1—N2—C3	122.0 (4)	C4A—S8A—C7A	91.6 (5)
C1—N2—Pd1	113.9 (3)	C5A—C4A—C3	125.9 (10)
C3—N2—Pd1	124.0 (3)	C5A—C4A—S8A	110.1 (9)
C14—N9—C10	119.2 (4)	C3—C4A—S8A	123.9 (8)
C14—N9—Pd1	128.1 (3)	C4A—C5A—C6A	113.9 (11)
C10—N9—Pd1	112.7 (3)	C4A—C5A—H5A	123.2
N2—C1—C10	118.8 (4)	C6A—C5A—H5A	122.9
N2—C1—H1	120.6	C7A—C6A—C5A	110.7 (11)
C10—C1—H1	120.6	C7A—C6A—H6A	124.7
C4B—C3—N2	117.9 (10)	C5A—C6A—H6A	124.7
N2—C3—C4A	116.2 (8)	C6A—C7A—S8A	113.7 (9)
C4B—C3—H3A	108.0	C6A—C7A—H7A	123.2
N2—C3—H3A	107.8	S8A—C7A—H7A	123.1
C4A—C3—H3A	109.4	C4B—S8B—C7B	91.3 (7)
C4B—C3—H3B	107.6	C5B—C4B—C3	126.5 (11)
N2—C3—H3B	107.8	C5B—C4B—S8B	111.5 (9)
C4A—C3—H3B	108.1	C3—C4B—S8B	122.0 (10)
H3A—C3—H3B	107.2	C4B—C5B—C6B	112.6 (11)
N9—C10—C11	120.6 (5)	C4B—C5B—H5B	123.7
N9—C10—C1	113.9 (4)	C6B—C5B—H5B	123.7
C11—C10—C1	125.6 (5)	C7B—C6B—C5B	111.1 (12)
C10—C11—C12	120.2 (5)	C7B—C6B—H6B	124.5
C10—C11—H11	119.9	C5B—C6B—H6B	124.4
C12—C11—H11	119.9	C6B—C7B—S8B	113.5 (11)
C13—C12—C11	118.8 (5)	C6B—C7B—H7B	123.2
C13—C12—H12	120.6	S8B—C7B—H7B	123.2
C11—C12—H12	120.6		
			
N9—Pd1—N2—C1	−1.8 (3)	Pd1—N9—C14—C13	−179.1 (4)
Cl16—Pd1—N2—C1	177.6 (3)	C12—C13—C14—N9	2.5 (8)
N9—Pd1—N2—C3	−178.8 (4)	C4B—C3—C4A—C5A	−50 (37)
Cl16—Pd1—N2—C3	0.6 (4)	N2—C3—C4A—C5A	112.1 (13)
N2—Pd1—N9—C14	−179.2 (4)	C4B—C3—C4A—S8A	126 (39)
Cl15—Pd1—N9—C14	1.4 (4)	N2—C3—C4A—S8A	−71.9 (14)
N2—Pd1—N9—C10	2.5 (3)	C7A—S8A—C4A—C5A	−1.8 (10)
Cl15—Pd1—N9—C10	−176.8 (3)	C7A—S8A—C4A—C3	−178.4 (14)
C3—N2—C1—C10	177.8 (4)	C3—C4A—C5A—C6A	178.5 (15)
Pd1—N2—C1—C10	0.7 (6)	S8A—C4A—C5A—C6A	2.0 (13)
C1—N2—C3—C4B	13.7 (10)	C4A—C5A—C6A—C7A	−1.0 (14)
Pd1—N2—C3—C4B	−169.5 (8)	C5A—C6A—C7A—S8A	−0.4 (13)
C1—N2—C3—C4A	13.0 (8)	C4A—S8A—C7A—C6A	1.3 (11)
Pd1—N2—C3—C4A	−170.2 (6)	N2—C3—C4B—C5B	−74 (2)
C14—N9—C10—C11	−1.0 (7)	C4A—C3—C4B—C5B	−56 (37)
Pd1—N9—C10—C11	177.4 (4)	N2—C3—C4B—S8B	106.1 (15)
C14—N9—C10—C1	178.7 (4)	C4A—C3—C4B—S8B	124 (39)
Pd1—N9—C10—C1	−2.9 (5)	C7B—S8B—C4B—C5B	0.0 (14)
N2—C1—C10—N9	1.5 (7)	C7B—S8B—C4B—C3	179.7 (18)
N2—C1—C10—C11	−178.7 (5)	C3—C4B—C5B—C6B	−179.9 (19)
N9—C10—C11—C12	1.5 (8)	S8B—C4B—C5B—C6B	−0.2 (15)
C1—C10—C11—C12	−178.2 (5)	C4B—C5B—C6B—C7B	0.4 (15)
C10—C11—C12—C13	0.0 (8)	C5B—C6B—C7B—S8B	−0.4 (17)
C11—C12—C13—C14	−2.0 (8)	C4B—S8B—C7B—C6B	0.2 (16)
C10—N9—C14—C13	−1.0 (7)		

### Hydrogen-bond geometry (Å, °)

**Table:**

*D*—H···*A*	*D*—H	H···*A*	*D*···*A*	*D*—H···*A*
C12—H12···Cl15^i^	0.95	2.70	3.508 (6)	143
C6B—H6B···Cl16^ii^	0.95	2.74	3.622 (14)	155
C7A—H7A···S8A^iii^	0.95	2.69	3.468 (12)	139

Symmetry codes: (i) −*x*+1, *y*+1/2, −*z*+3/2; (ii) −*x*, *y*+1/2, −*z*+1/2; (iii) *x*, −*y*+1/2, *z*−1/2.

## References

[bb1] Atwood, J. L. & Barbour, L. J. (2003). *Cryst. Growth Des.* **3**, 3–11.

[bb2] Barbour, L. J. (2001). *J. Supramol. Chem.* **1**, 189–191.

[bb3] Bianchini, C., Giambastiani, G., Luconi, L. & Meli, A. (2010). *Coord. Chem. Rev.* **254**, 431–455.

[bb4] Britovsek, G. J. P., Baugh, S. P. D., Hoarau, O., Gibson, V. C., Wass, D. F., White, A. J. P. & Williams, D. J. (2003). *Inorg. Chim. Acta*, **345**, 279–291.

[bb5] Bruker (2009). *SMART*, *SAINT* and *SADABS* Bruker AXS Inc., Madison, Wisconsin, USA.

[bb6] Doherty, S., Knight, J. G., Scanlam, T. H., Elsegood, M. R. J. & Clegg, W. (2002). *J. Organomet. Chem.* **650**, 231–248.

[bb7] Motswainyana, W. M., Ojwach, S. O., Onani, M. O., Iwuoha, E. I. & Darkwa, J. (2011). *Polyhedron*, **30**, 2574-2580.

[bb8] Ojwach, S. O., Guzei, I. A. & Darkwa, J. (2009). *J. Organomet. Chem.* **694**, 1393–1399.

[bb9] Sheldrick, G. M. (2008). *Acta Cryst.* A**64**, 112–122. 10.1107/S0108767307043930 18156677

[bb10] Zhang, W., Sun, W.-H., Wu, B., Zhang, S., Ma, H., Li, Y., Cheng, J. & Hao, P. (2006). *J. Organomet. Chem.* **691**, 4759–4767.

